# Development of Substrate Degradation Enzyme Therapy for Mucopolysaccharidosis IVA Murine Model

**DOI:** 10.3390/ijms20174139

**Published:** 2019-08-24

**Authors:** Kazuki Sawamoto, Shunji Tomatsu

**Affiliations:** 1 Nemours/Alfred I. duPont Hospital for Children, 1600 Rockland Rd., Wilmington, DE 19803, USA; 2 Department of Pediatrics, Graduate School of Medicine, Gifu University, Gifu 501-1193, Japan; 3 Department of Pediatrics, Thomas Jefferson University, Philadelphia, PA 19107, USA

**Keywords:** MPS IVA, GALNS, thermostable keratanase, keratan sulfate, skeletal dysplasia

## Abstract

Mucopolysaccharidosis IVA (MPS IVA) is caused by a deficiency of the lysosomal enzyme N-acetylgalactosamine-6-sulfate sulfatase (GALNS). Conventional enzyme replacement therapy (ERT) is approved for MPS IVA. However, the fact that the infused enzyme cannot penetrate avascular lesions in cartilage leads to minimal impact on the bone lesion. Moreover, short half-life, high cost, instability, and narrow optimal pH range remain unmet challenges in ERT. Thermostable keratanase, endo-β-*N*-acetylglucosaminidase, has a unique character of a wide optimal pH range of pH 5.0–7.0. We hypothesized that this endoglycosidase degrades keratan sulfate (KS) polymer in circulating blood and, therefore, ameliorates the accumulation of KS in multiple tissues. We propose a novel approach, *Substrate Degradation Enzyme Therapy (SDET),* to treat bone lesion of MPS IVA. We assessed the effect of thermostable keratanase on blood KS level and bone pathology using *Galns* knock-out MPS IVA mice. After a single administration of 2 U/kg (= 0.2 mg/kg) of the enzyme at 8 weeks of age via intravenous injection, the level of serum KS was significantly decreased to normal range level, and this suppression was maintained for at least 4 weeks. We administered 2 U/kg of the enzyme to MPS IVA mice every fourth week for 12 weeks (total of 3 times) at newborns or 8 weeks of age. After a third injection, serum mono-sulfated KS levels were kept low for 4 weeks, similar to that in control mice, and at 12 weeks, bone pathology was markedly improved when SDET started at newborns, compared with untreated MPS IVA mice. Overall, thermostable keratanase reduces the level of KS in blood and provides a positive impact on cartilage lesions, demonstrating that SDET is a novel therapeutic approach to MPS IVA.

## 1. Introduction

Mucopolysaccharidosis type IVA (MPS IVA, also called Morquio Syndrome type A; OMIM 253000) is an autosomal recessive disorder caused by a deficiency of the lysosomal enzyme N-acetylgalactosamine-6-sulfate sulfatase (GALNS) [1,2,3,4,5,6]. GALNS involves the degradation of glycosaminoglycans (GAG), keratan sulfate (KS), and chondroitin-6-sulfate (C6S). Deficiency of this enzyme causes progressive accumulation of KS and C6S in systemic tissues, especially cartilage and its extracellular matrix (ECM), leading to a devastating skeletal dysplasia with incomplete ossification and successive imbalance of growth [4,7,8]. Clinical features of skeletal dysplasia in MPS IVA patients include short neck, pectus carinatum, cervical spinal cord compression, laxity of joints, kyphoscoliosis, coxa valga, disproportionate short trunk, and genu valgum. Airway compromise (tracheal obstruction), spinal cord compression, and cardiovascular involvements are life-threatening problems to severe type of MPS IVA patients [4,7,9,10,11].

Enzyme replacement therapy (ERT) and hematopoietic stem cell transplantation (HSCT) coupled with various surgical interventions are treatment options currently available for patients with MPS IVA in clinical practice. ERT and HSCT are based on the principle of cross-correction that lysosomal enzymes are uptaken into cells via the mannose-6-phosphate receptor. ERT for MPS IVA was approved in 2014 by the Food and Drug Administration; however, several limitations are indicated: (1) weekly infusions for 5–6 h, (2) high cost [12,13], and (3) rapid clearance (a short half-life time, 35 min) [14]. In addition, patients treated with ERT have not shown any improvement in hypermobile joints, skeletal dysplasia, or bone pathology [11,15,16,17], as observed in MPS IVA mice treated with ERT [18,19]. Thus, there is no proof that the current ERT provides an impact on bone pathology in MPS IVA, even after long-term treatment [15,16,19]. Our study has shown that ERT did not improve the accumulation of storage materials in chondrocytes from surgical remnants during 6–60 months of this therapy [11,15,16]. Moreover, even if ERT started in a severe type of patients with MPS IVA before two years of age, Do Cao et al. and we did not observe the improvement of the bone growth and actual height [17,20].

HSCT for MPS IVA was approved in Japan in 1995 with the initial success of transplantation, and at least four patients have been followed over 15–25 years [21]. Only one patient underwent one surgical intervention during the follow-up years. HSCT may provide a better outcome than ERT in patients with MPS IVA. Dr. Wang et al. and we have monitored 13 cases of MPS IVA patients treated with HSCT and have demonstrated the improvement of pulmonary function, the activity of daily living, bone mineral density, cardiovascular function, and laxity of joints, as well as reduction of the number of the surgical procedures [15,21,22,23,24]. However, HSCT has several critical issues: (1) finding the appropriate donor, (2) risks of graft versus host disease and rejection, (3) limited impact on bone lesions, and (4) requirement of well-trained staffs and facilities. Therefore, effective and feasible therapy for a bone lesion in MPS IVA is an unmet challenge.

In addition, recent studies have indicated that not only accumulated GAG within cells but that in the neighboring ECM provide a critical role to lead to abnormal chondrogenesis and endochondral ossification [25,26,27,28]. Degradation of KS occurs in a step-wise manner by removal or processing of the terminal residue on the non-reducing end of this polymer chain, involving various enzymes including GALNS, *N*-acetylglucosamine 6-sulfatase, β-*N*-acetylhexosaminidase, and β-galactosidase. These exohydrolases degrade GAG in lysosomes within the cell since the lysosomal enzymes are active only in an acidic environment with narrow pH range from 4.5 to 5.0. Current ERT does not directly degrade accumulated KS in blood and ECM in multiple tissues, including bone and cartilage lesions.

We have purified a KS hydrolase, endo-β-*N*-acetylglucosaminidase from *Bacillus circulans* and have identified a characterization of thermostability in this enzyme. Thus we named this enzyme, thermostable keratanase [29]. Thermostable keratanase is uniquely active and stable over a broad physiological pH range (pH: 5.0–7.0). Here, we propose a novel enzyme therapy by using thermostable keratanase, a bacterial endo-β-*N*-acetylglucosaminidase, which can directly degrade KS polymer in blood circulation. We have hypothesized that the elimination of KS polymer from the circulating blood using thermostable keratanase consequently ameliorates the accumulation of KS in multiple tissues and their ECM.

In this study, we have investigated the therapeutic efficacy of thermostable keratanase in skeletal abnormality using MPS IVA mice. We termed our novel, innovative therapy as “*substrate degradation enzyme therapy* (SDET).”

## 2. Results

### 2.1. Production, Purification, and Characterization of Thermostable Keratanase

**Figure 1:**
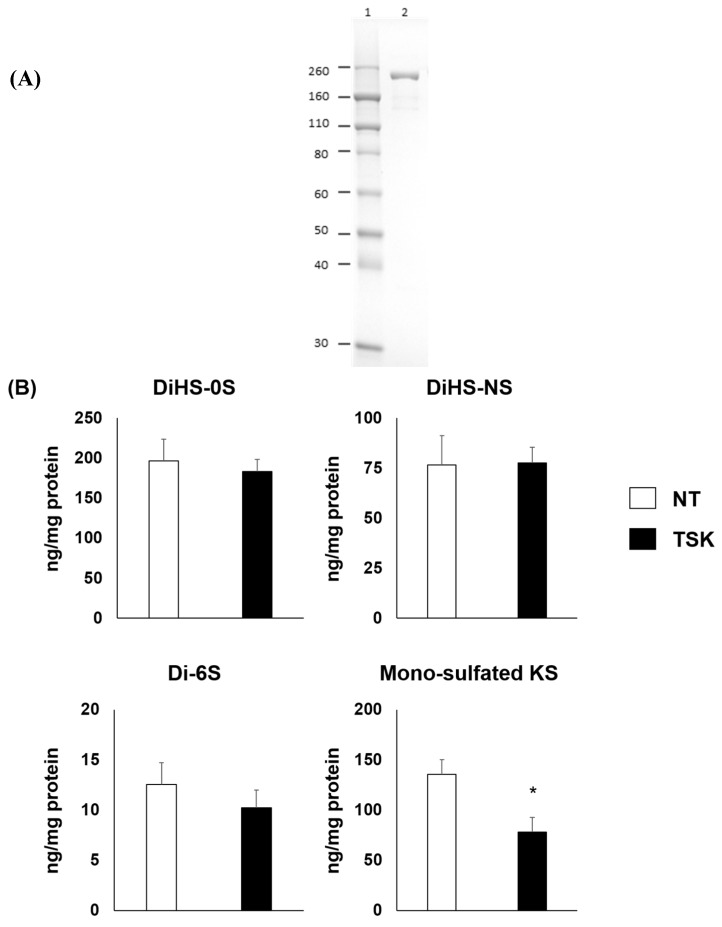
Purification and characterization of the thermostable keratanase. (**A**) SDS-PAGE of thermostable keratanase. The purified thermostable keratanase was applied to a 10% Tris-Glycine SDS-PAGE gel, and the proteins were visualized by Coomassie Brilliant Blue G-250 staining. Lane 1, markers; lane 2, purified enzyme (5 mU, 0.4 μg). (**B**) Treatment with 1 μU/mL purified thermostable keratanase degraded KS chains but not diHS-0S, diHS-NS and C6S chains in MPS IVA chondrocyte cells from 3-dimensional (3D) culture; *n* = 3. Statistics were analyzed by unpaired *t*-test. Data are presented as mean ± SD. * *p* < 0.05. C6S: chondroitin 6 sulfate, HS: heparan sulfate, KS: keratan sulfate, NT: untreated, TSK: thermostable keratanase.

The thermostable keratanase was purified from *B. circulans* cell body as described in materials and methods. Total 450 U of keratanase was obtained using 3 steps of column chromatography. SDS-PAGE analysis of the enzyme showed a single band corresponding to a *M_r_* of 220,000 (Figure 1A). The finally purified enzyme activity was 50.0 U/mL; the specific activity was 11.5 U/mg protein, and endotoxin level was 12.8 Endotoxin Unit/mg protein. The optimum pH of the enzyme was pH 5–7 [29]. Enzyme activity was stable for 120 h at 37 °C [30].

### 2.2. In Vitro Efficacy Study of Thermostable Keratanase

The 3-D culture of chondrocyte cells derived from MPS IVA patient showed the excessive accumulation of GAG. After treatment of 1 μU/mL thermostable keratanase, the level of mono-sulfated KS significantly decreased compared to the untreated group. In contrast, the levels of other GAG, diHS-0S, diHS-NS, and di-6S (dermatan sulfate) were unchanged (Figure 1B).

### 2.3. Preliminary Toxicity Study of Thermostable Keratanase in Mice

To assess the toxicity of thermostable keratanase, groups of 6 male C57BL/6J mice received weekly intravenous injections of 250 U/kg of thermostable keratanase for a total of 4 times. No mice died during the experimental period, and no treatment-related changes were noted in body weight loss. In the clinical observations on the day of each treatment, rapid shallow breathing, and/or decreased locomotor activity were seen sporadically 0 to 40 min after receiving thermostable keratanase. These findings were transient and most mice recovered within 10 min.

### 2.4. Serum and Tissue Levels of KS in MPS IVA Mice after a Single Injection of Thermostable Keratanase

**Figure 2:**
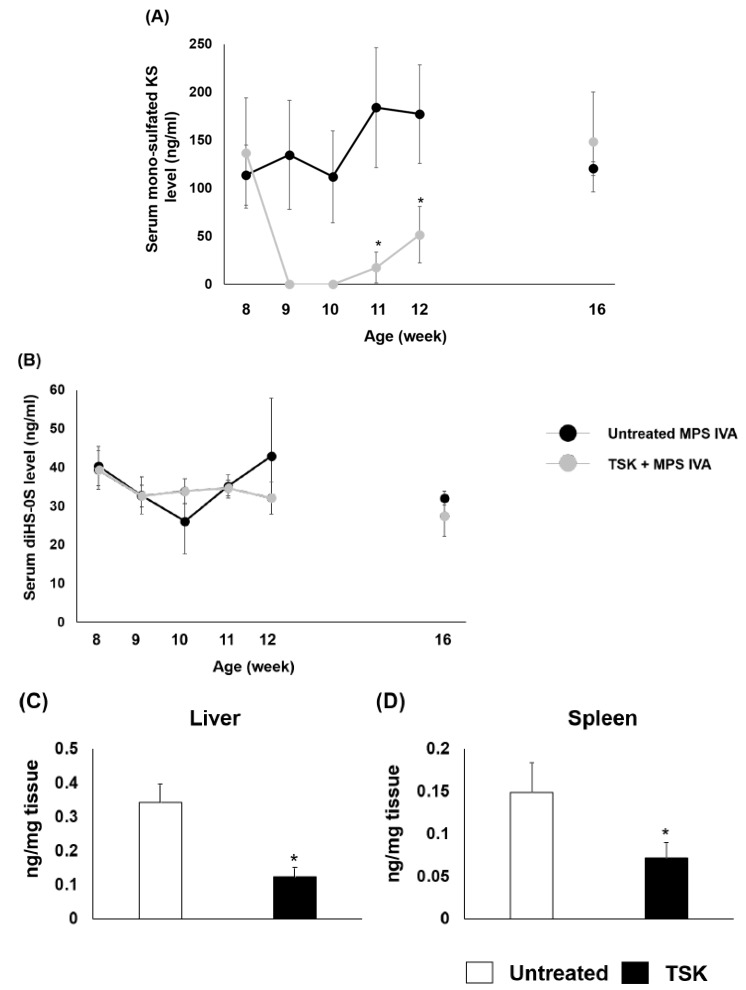
Serum and tissue glycosaminoglycan levels of MPS IVA mice after a single administration of thermostable keratanase. MPS IVA mice at 8 weeks of age were treated with 2 U/kg of thermostable keratanase intravenously, and PBS was administered in the untreated group. Serum samples were collected from the superficial temporal vein at the time points of 8, 9, 10, 11, 12, and 16 weeks of age. The level of mono-sulfated KS (**A**) and diHS-0S (**B**) was measured by LC-MS/MS; *n* = 4–5. The tissue sample was collected from MPS IVA mice 24 h post-injection of thermostable keratanase, and the levels of mono-sulfated KS in liver (**C**) and spleen (**D**) were measured by LC-MS/MS; *n* = 4. Statistics were analyzed by unpaired t-test. Data are presented as mean ± SD. * *p* < 0.05 vs. untreated MPS IVA. HS: heparan sulfate, KS: keratan sulfate, TSK: thermostable keratanase.

To confirm the activity of this endohydrolase in mice, MPS IVA mice received a single intravenous injection of 2 U/kg of thermostable keratanase at 8 weeks of age. Serum KS and diHS-0S levels were monitored at weeks 0 (before treatment), 1, 2, 3, 4, and 8. The amounts of mono-sulfated KS in the liver and spleen were measured 24 h post-injection. The levels of serum mono-sulfated KS were undetectable at 1 to 2 weeks post-injection, and the levels were maintained low for at least 4 weeks post-injection (Figure 2A). In contrast, the levels of serum diHS-0S were similar between untreated and treated mice during this study (Figure 2B). The amount of mono-sulfated KS in the liver and spleen of MPS IVA mice were decreased in treated mice. (Figure 2C,D).

### 2.5. Therapeutic Effects of Thermostable Keratanase in MPS IVA Mice

To evaluate the efficacy of thermostable keratanase in bone and cartilage lesions, MPS IVA mice were treated with 2 U/kg of the enzyme intravenously every 4 weeks for a total of 3 times from neonatal period to 12 weeks of age (newborn administration study).

**Figure 3:**
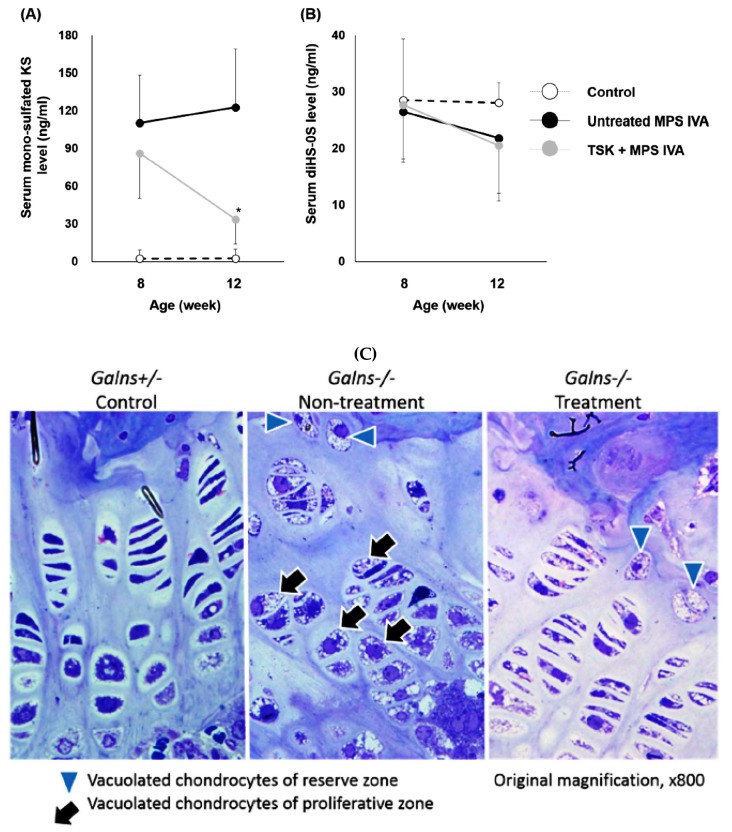
Serum glycosaminoglycan levels and bone pathology of MPS IVA mice after repeated administration of thermostable keratanase. MPS IVA mice were treated with 2 U/kg of thermostable keratanase intravenously 3 times at 0, 4, and 8 weeks of age, and PBS was administered into MPS IVA mice (untreated) and heterozygous mice (control) in the same manner. Serum samples were collected from the superficial temporal vein at the time points of 8 and 12 weeks of age. Tissues were collected at 12 weeks of age. (**A**,**B**) The level of serum mono-sulfated KS and diHS-0S was measured by LC-MS/MS. *n* = 7–9. (**C**) Bone pathology in growth plate regions of femur and tibia was evaluated by toluidine blue staining. Statistics were analyzed by one-way ANOVA with the Bonferroni’s post-hoc test. Data are presented as mean ± SD. *p < 0.05 vs. untreated MPS IVA. HS: heparan sulfate, KS: keratan sulfate, TSK: thermostable keratanase.

In the neonatal administration study, serum levels of mono-sulfated KS in treated mice moderately decreased, compared to those in untreated MPS IVA mice at 8 weeks of age and significantly decreased at 12 weeks of age (Figure 3A). Serum diHS-0S levels were similar between untreated and treated mice at 8 and 12 weeks of age (Figure 3B). The histopathologic scores in the treated group were lower than in the untreated group at 12 weeks of age (Figure 3C). Severe vacuolization of chondrocytes (score 3) was noted in 67% of the untreated group, while in 29% of the treated group. Moderate to minimal disorganization of chondrocytes (score 2-1) was noted in 56%–33% of the untreated group, while in 43%–14% of the treated group (Table 1).

## 3. Discussion

The goal of this study was to evaluate the therapeutic efficacy of thermostable keratanase, a bacterial endoglycosidase, in MPS IVA mice. We have demonstrated that thermostable keratanase into MPS IVA mice provided an impact on (i) a significant reduction (normalization) in levels of serum KS and (ii) a decrease in vacuolation of proliferative zone chondrocytes in the growth plates. Thus, thermostable keratanase showed therapeutic efficacy in cartilage, in which ERT has shown very limited efficacy [18,19,31]. After a single administration of thermostable keratanase to MPS IVA mice at 8 weeks of age, the levels of serum KS were immediately decreased and normalized. This reduction of KS suggests that thermostable keratanase directly clears KS in the blood since this enzyme has a wide optimal pH range from 5.0–7.0. This KS reduction was maintained for at least 4 weeks; indeed, every 4 weeks for a total of 3 times administration of thermostable keratanase resulted in a lower degree of vacuolization in the chondrocytes in the treated group than that of the untreated group in the neonatal administration study.

On the other hand, our previous study in MPS IVA mice treated with recombinant human GALNS showed that the levels of KS in blood were not normalized to the wild type level even after 12 weekly ERT [18]. Overall, these findings demonstrate that SDET is superior to ERT in not only therapeutic efficacy but the less frequency of administration.

Additionally, current ERT degrades accumulated GAGs only in lysosomes within the cells but not accumulated GAGs in ECM due to its optimal acidic pH. There is, however, a possibility that thermostable keratanase penetrated the ECM of the cartilage and directly degraded the accumulated KS as with the enzyme directly degraded KS in the circulating blood. In fact, we have found that KS levels in bone and other tissues were significantly reduced compared with untreated MPS IVA mice after thermostable keratanase twice infusions at day 0 or 1 and 2 weeks old. This suggests that KS in ECM has been degraded (in progress, data not shown). Further study of the tissue distribution of administered thermostable keratanase is required to confirm this possibility.

Recent studies have shown that inflammation plays an important role in the pathophysiology of MPS. The fragments of excessive GAG released in ECM stimulate toll-like receptor 4 (TLR4) [32,33]. Activation of NF-κB signaling via TLR4 resulted in the production of various inflammatory cytokine and protease such as TNF-α and MMP. Simonaro et al. demonstrated that inactivation of TLR4 in MPS VII mice reduced serum TNF-α levels and provided a significant positive effect on the orientation of column structure in their growth plate regions, leading to improvement of bone length [26]. Moreover, this group also reported that an anti-inflammatory drug, pentosan polysulfate, improved column structure in the growth plate although storage materials remained in chondrocytes [28]. These data suggest that GAG accumulation in ECM and successive inflammation in cartilage leads to worsening skeletal dysplasia in MPS. Immunohistochemical staining showed that total proteoglycans were highly accumulated in ECM from the femoral condyle of the knee in a severe type of MPS IVA patient, compared to that in healthy controls [34]. Therefore, it is likely that administration of endoglycosidase, thermostable keratanase, degrades and removes KS in ECM as well as the circulating blood, more directly than GALNS and consequently improves bone abnormality in MPS IVA via some anti-inflammatory effects.

We have developed a sensitive and quantitative method to measure KS in blood and urine from human by LC-MS/MS, which can identify two types of disaccharide KS (mono-sulfated KS and di-sulfated KS) [35]. The levels of KS in blood and urine correlate with the clinical severity of patients with MPS IVA [4,36,37,38]. However, patients with MPS IVA treated with ERT did not show any improvement in the bone lesion, although the level of urine KS was substantially decreased. In contrast, blood KS levels remained unchanged [4,39,40]. Urine and blood GAG, including KS, are likely to come from a different origin [41,42].

In this study, we found that MPS IVA mice have significantly high levels of mono-sulfated KS in blood, compared to heterozygous mice. We have monitored mono-sulfated KS level in the blood, correlating with the improvement of bone pathology in MPS IVA mice. Therefore, blood KS levels may be a more useful biomarker to evaluate treatment efficacy on bone lesions of these patients than urinary KS levels.

In this study, we administered 2 U/kg of thermostable keratanase into MPS IVA mice three times, resulting in the improvement of the bone pathology at the growth plate region when we started treatment of newborns. To maximize therapeutic efficacy of thermostable keratanase, further studies are required with consideration of the following factors: (i) dose-dependent administration, (ii) age-dependent administration, and (iii) frequency and duration of administration. A higher dose than 2 U/kg would be allowed since no apparent toxicity was noted in the preliminary toxicity study. We need to consider that the use of a non-mammalian enzyme may evoke a serious immune response. Development of immunosuppression strategies will be required, such as biodegradable biomaterials (like PEGylation) encapsulating the enzyme. A PEGylated drug derived from a bacterial enzyme is approved for phenylketonuria. This drug affected decreasing blood phenylalanine levels but produced IgG and IgM antibodies against PEG [43]. Other immunosuppressing procedures still should be considered.

It is notable that the proof of therapeutic efficacy with thermostable keratanase in MPS IVA mice indicates that other bacterial GAG endoglycosidases such as chondroitinase ABC, chondroitinase B, or heparinase would be applicable to treat other types of MPS. Successful completion of further study should provide a significant impact on the MPS field.

In conclusion, administration of thermostable keratanase reduced levels of serum KS and ameliorated the skeletal abnormalities of MPS IVA mice. Our novel, innovative therapy “SDET” provides a potential approach to break down KS in the bloodstream and possibly the ECM and consequently clears the accumulation of KS in multiple tissues, including cartilage, the region that ERT has little effect on.

## 4. Materials and Methods

### 4.1. Production of Thermostable Keratanase from *Bacillus circulans* KsT202

Thermostable keratanase was extracted from *B. circulans* as previously described [29]. Briefly, KsT202 was inoculated in medium (1% peptone, 0.75% Brewery’s yeast extract, 0.25% fish meal extract, 0.5% KH_2_PO_4_, 0.02% MgSO_4_·7H_2_O, 0.1% NaCl, 150 ppm Adecanol LG-109, and 0.5% shark cartilage KS, pH7.5), and cultured at 37 °C. Two hundred grams of frozen bacteria pellets obtained from 20 L of culture was resuspended in 1 L of 100 mM phosphate buffer, pH7.2 and treated with both lysozyme (50 μg/mL) and DNase (4 μg/mL) for 60 min at 37 °C. Then 3 L of cold 33 mM phosphate buffer was added and centrifuged. The supernatant was collected and applied to a DEAE cellulose DE52 column (Whatman, Maidstone, UK) equilibrated with 10 mM Tris-HCl, pH7.2. The enzyme fraction was eluted up to 0.4 M NaCl gradient. After the addition of NaCl to a final concentration of 4 M, the enzyme fraction was applied to a phenyl sepharose 6 fast flow column (GE Healthcare, IL, US), equilibrated with the 10 mM phosphate buffer, 4 M NaCl, pH7.2. The enzyme fraction was eluted using 4 to 0 M NaCl linear gradient, followed by dialyzation against 100 mM sodium acetate, pH6.0. To remove endotoxin, the enzyme was applied to an Endo Trap HD column (Hyglos GmbH, Munich, Germany) equilibrated with 0.02 M HEPES, 0.15 M NaCl and 0.1 mM CaCl_2_, pH7.4. Then, flow-through fractions were applied to the regenerated Endo Trap HD column repeatedly. Finally, the enzyme fraction was buffer exchanged with ultrafiltration to phosphate buffered saline (PBS) and stored at under −80 °C until use.

### 4.2. Keratanase Assay, Endotoxin Assay, and Protein Assay

The keratanase assay was carried out according to the method of Park and Johnson [44] and performed as previously described [29]. The endotoxin concentration was measured by Endospecy ES-50M (Seikagaku Corp., Tokyo, Japan). The protein concentration was measured by Lowry assay or micro BCA assay (Thermo Fisher Scientific, Waltham, MA, USA). Bovine serum albumin was used as a reference standard.

### 4.3. 3-Dimensional Chondrocyte Cell Culture

Chondrocyte cells derived from a MPS IVA patient was maintained at 37 °C in a 5% CO_2_ incubator in CBM™ Basal Medium containing CGM™ SingleQuots™ Kit (chondrocyte culture medium) (Lonza, Walkersville, MD, USA). The 3-dimensional (3-D) chondrocyte cell culture was followed according to the supplier’s instruction. Briefly, the cells were diluted with 1.2% sodium alginate solution, and the cell suspension was dropped into CaCl_2_ solution in 6-well plate with a 22G needle. The dropped beads were washed by 155 mM NaCl several times and incubated in chondrocyte growth medium for 28 days. Then, the cells were treated with 1 μU/mL thermostable keratanase for 72 h incubation. After the end of incubation, the beads were depolymerized with 55 mM sodium citrate solution and washed with PBS 3 times. The cell pellets were sonicated on ice and stored at −20 °C until processing for GAG assay.

### 4.4. Preliminary Toxicity Study of Thermostable Keratanase in Mice

C57BL/6J mice (6 males) at 6–7 weeks of age were weekly treated with 250 U/kg of thermostable keratanase via a tail vein and monitored until day 25. As for control groups, 3 male mice were treated with saline. The behavioral signs of mice were observed until 1 h after administration. All animal cares and experiments were approved by the Institutional Animal Care and Use Committee of Seikagaku Corporation and carried out by relevant guidelines and regulations.

### 4.5. Intravenous Injection of Thermostable Keratanase to MPS IVA Mice

The generation of MPS IVA knockout (KO) mouse (*Galns^−/−^*) in C57BL/6 background was described previously [45]. Genotype was determined by PCR on day 14. Three treatment studies were performed. (1) In single administration study, MPS IVA mice at 8 weeks of age received a single intravenous injection of 2 U/kg thermostable keratanase via the tail vein. Serum KS and diHS-0S levels were monitored at weeks 0 (before treatment), 1, 2, 3, 4, and 8. Untreated mice received PBS in the same manner. Blood samples were collected from the superficial temporal vein at the time points of 8 (before treatment), 9, 10, 11, 12, and 16 weeks of age. (2) In the neonatal administration study, at 1 or 2 days after birth, neonatal MPS IVA mice were treated with 2 U/kg thermostable keratanase via a superficial temporal vein and injected with 2 U/kg of this enzyme intravenously at 4 and 8 weeks of age and monitored until 12 weeks of age. PBS was administered into MPS IVA mice (untreated) and heterozygous mice (control) in the same manner. Blood samples were collected from the superficial temporal vein at the time points of 8 and 12 weeks of age. Tissues were collected at 12 weeks of age. (3) In the tissue levels of KS measurement study, MPS IVA mice were treated with 2U/kg thermostable keratanase intravenously at 4 weeks of age, and tissues were collected after 24 h of administration. At each time point, approximately 100 μL of blood was collected in separator tubes (BD, Franklin Lakes, NJ, USA). The blood was centrifuged at 8000 rpm for 10 min, and serum separated was kept at −20 °C until performing GAG assay by LC-MS/MS. At necropsy, mice were euthanized in a CO_2_ chamber and perfused with 20 mL of 0.9% saline. Liver and spleen were collected and stored at −80 °C until processing for GAG assay. Bone tissues were stored in 10% neutral buffered *formalin* until histological processing. All animal cares and experiments were approved by the Institutional Animal Care and Use Committee of Nemours/Alfred I. duPont Hospital for Children (Protocol#: NBR 2014-003, Date: 26-3-2014).

### 4.6. Extraction of GAG from Tissue

We previously developed the GAG extraction method from various tissues in mice [46]. Briefly, dissected liver and spleen were frozen in liquid nitrogen and homogenized with acetone using a homogenizer. After centrifugation, the obtained tissue acetone powder was dried up under centrifugal vacuum concentrator. The defatted tissue was suspended in 0.5 M NaOH and incubated at 50 °C for 2 h to remove GAG polymers from its proteoglycan core protein. After neutralization with 1 M HCl, NaCl was added to a final concentration of 3 M. Insoluble materials were removed by centrifugation, and the pH of the supernatant was adjusted below 1.0 with 1 M HCl. The precipitate was removed by centrifugation, and the supernatant was neutralized with 1 M NaOH. The GAG was precipitated by the addition of two volumes of ethanol containing 1.3% potassium acetate. After centrifugation, the precipitated pellet was washed by 80% cold ethanol and dissolved in distilled water.

### 4.7. GAG Assay

Serum and tissue GAG level were measured by LC-MS/MS as described previously [35,47,48,49,50]. Briefly, serum and homogenated tissues were filtered by 96 well omega 10K filter plate (Pall Corporation, Port Washington, NY, USA). Mixture solution containing 5 μg/mL internal standard (IS), 1 mU heparitinase, 1 mU chondroitinase ABC and 1 mU keratanase II was added to the filter plate, and samples were incubated at 37 °C water bath overnight. Then, the samples were centrifuged for 15 min at 2500× *g*, and 10 μL of filtered samples were injected to the apparatus consisted of a 1290 Infinity LC system with a 6460 triple quad mass spectrometer (Agilent Technologies, Palo Alto, CA, USA). Each disaccharide was separated on a Hypercarb column (2.0 mm i.d. 50 mm length; 5 μm particles; Thermo Fisher Scientific, Waltham, MA, USA), maintained at 60°C. The mobile phase was a gradient elution of 5 mM ammonium acetate, pH 11.0 (solvent A) to 100% acetonitrile (solvent B). The flow rate was 0.7 mL/min, and the gradient was as follows: 0 min 100% solvent A, 1 min 70% solvent A, 2 min 70% solution A, 2.20 min 0% solvent A, 2.60 min 0% solvent A, 2.61 min 100% solvent A, 5 min 100% solvent A. The mass spectrometer was operated with electrospray ionization in the negative ion mode (Agilent Jet Stream technology). Each disaccharide was quantified by specific m/z precursor and product ions [IS, 354.3→193.1; mono-sulfated KS, 462→97; diHS-0S 378.3→175.1; diHS-NS 416.0→138.1; Di-6S 458.4→282.1]. Chondrosine was used as an IS.

### 4.8. Histopathological Staining

Toluidine blue staining and hematoxylin and eosin staining were performed as previously described [51]. Briefly, knee joint (femur and tibia) was collected from MPS IVA mice at 12 weeks old to evaluate the therapeutic effects of thermostable keratanase by light microscopy. For toluidine blue staining, tissues were fixed in 4% glutaraldehyde, 2% paraformaldehyde in PBS, and post-fixed in osmium tetroxide and embedded in Spurr’s resin. Then, toluidine blue-stained 0.5-µm-thick sections were prepared. For hematoxylin and eosin staining, tissues were fixed in 10% neutral buffered formalin and embedded in paraffin. Then, hematoxylin and eosin stained 3-µm-thick sections were prepared. Histopathological findings were evaluated between the controls and treatment groups. The degrees of chondrocyte vacuolation and chondrocyte alignment of the growth plates were scored. “None” was score 0 (−), “minimal” was score 1 (+), “moderate” was score 2 (++), and “severe” was score 3 (+++).

### 4.9. Statistical Analysis

All data were expressed as means and standard deviations (SD). Two comparison tests and multiple comparison tests were performed by unpaired t-test or one-way ANOVA with the Bonferroni’s post-hoc test using GraphPad Prism 5.0 (GraphPad Software, San Diego, CA, USA), respectively. The statistical significance of difference was considered as *p* < 0.05.

## Figures and Tables

**Table 1:** Pathological score in growth plate region of femur/tibia of MPS IVA mouse.

	Score 0 (None)	Score 1 (Minimal)	Score 2 (Moderate)	Score 3 (Severe)	Total Mouse *n*
(a) Vacuolization of chondrocyte					
Control	100% (8/8)	0% (0/8)	0% (0/8)	0% (0/8)	8
Untreated	0% (0/9)	0% (0/9)	33% (3/9)	67% (6/9)	9
Treated	0% (0/7)	14% (1/7)	57% (4/7)	29% (2/7)	7
(b) Disorganization of column structure					
Control	100% (8/8)	0% (0/8)	0% (0/8)	0% (0/8)	8
Untreated	11% (1/9)	33% (3/9)	56% (5/9)	0% (0/9)	9
Treated	43% (3/7)	14% (1/7)	43% (3/7)	0% (0/7)	7

(a) Vacuolization of chondrocyte in proliferative lesion: Score 0 (None), Not observed; Score 1 (Minimal), <20%; Score 2 (Moderate), 20%–50%; Score 3 (Severe), >50%. (b) Disorganization of column structure in proliferative lesion: Score 0 (None), Not observed; Score 1 (Minimal), <20%; Score 2 (Moderate), 20%–50%; Score 3 (Severe), >50%.

## Abbreviations

3-D	3-dimensional
C6S	chondroitin-6-sulfate
ECM	extracellular matrix
ERT	enzyme replacement therapy
GAG	glycosaminoglycans
GALNS	N-acetylgalactosamine 6-sulfate sulfatase
HS	heparan sulfate
HSCT	hematopoietic stem cell transplantation
IS	internal standard
KS	keratan sulfate
LC-MS/MS	liquid chromatography/tandem mass spectrometry
MPS	Mucopolysaccharidosis
PBS	phosphate buffered saline
SDET	substrate degradation enzyme therapy
TLR4	toll-like receptor 4
